# Hyperuniform disordered waveguides and devices for near infrared silicon photonics

**DOI:** 10.1038/s41598-019-56692-5

**Published:** 2019-12-30

**Authors:** Milan M. Milošević, Weining Man, Geev Nahal, Paul J. Steinhardt, Salvatore Torquato, Paul M. Chaikin, Timothy Amoah, Bowen Yu, Ruth Ann Mullen, Marian Florescu

**Affiliations:** 1 Zepler Institute for Photonics and Nanoelectronics, Optoelectronics Research Centre, University of Southampton, Southampton, SO17 1BJ UK; 2 Department of Physics and Astronomy, San Francisco State University, San Francisco, CA 94132 USA; 3 Department of Physics, Princeton University, Princeton, New Jersey 08544 USA; 4 Department of Chemistry, Princeton University, Princeton, New Jersey 08544 USA; 5 Department of Physics, New York University, New York, NY 10003 USA; 6 Department of Physics and the Advanced Technology Institute, University of Surrey, Guildford, GU2 7XH UK; 7 Etaphase, Incorporated, Seattle, WA USA

**Keywords:** Energy science and technology, Engineering, Materials science, Nanoscience and technology, Optics and photonics

## Abstract

We introduce a hyperuniform-disordered platform for the realization of near-infrared photonic devices on a silicon-on-insulator platform, demonstrating the functionality of these structures in a flexible silicon photonics integrated circuit platform unconstrained by crystalline symmetries. The designs proposed advantageously leverage the large, complete, and isotropic photonic band gaps provided by hyperuniform disordered structures. An integrated design for a compact, sub-volt, sub-fJ/bit, hyperuniform-clad, electrically controlled resonant optical modulator suitable for fabrication in the silicon photonics ecosystem is presented along with simulation results. We also report results for passive device elements, including waveguides and resonators, which are seamlessly integrated with conventional silicon-on-insulator strip waveguides and vertical couplers. We show that the hyperuniform-disordered platform enables improved compactness, enhanced energy efficiency, and better temperature stability compared to the silicon photonics devices based on rib and strip waveguides.

## Introduction

In the past three decades, broad worldwide academic and commercial efforts in silicon photonics have led to the realization of Terabit-scale optical data communications at increasingly lower-costs as required for the rapidly growing demand for interconnects between servers in data centers^1,2^. Explosive growth in cloud computing and entertainment-on-demand pose increasingly challenging cost, energy, and interconnect density requirements on data transmission, processing, and storage. Optical data links now replace traditional copper-based solutions in long haul, metro, and top-of-rack data center interconnect networks. These optical interconnects offer steadily increasing potential to minimize latency and power consumption while maximizing bandwidth, interconnect density, device density, and reliability. Silicon photonics leverages the large-scale complementary metal-oxide semiconductor (CMOS) manufacturing processes and facilities to produce high-performance optical transceivers with high yield at low cost^1–4^, making its application to optical transceivers increasingly compelling over shorter and shorter distances^5–8^.

Soref’s prescient identification of silicon as a promising material for photonic integration over three decades ago^9^ led to steady development and now-rapid production of increasingly-complex photonic integrated circuits (PICs) containing hundreds to thousands of components^10^ using the same CMOS lithography tools that enabled multi-decadal Moore’s Law growth in the semiconductor electronics industry. Wide-ranging applications of photonic integration including data communications, computing, and sensing all share a common need for compactness, sensitivity, and energy-efficiency. In particular, for interconnection of servers in data centers, the massively-parallel compact integration of large numbers of energy-efficient optical components on a single chip is increasingly important for the continued scaling of cloud computing applications ranging from search and advertising to deep learning, artificial intelligence and the internet of things^9–11^. The very high levels of integration afforded by silicon photonics substantially increase the functionality of individual chips, beneficially driving-down costs by minimizing both the number of components in each package and the number of packages on each board. The compactness of commercial silicon photonics systems based on conventional rib or strip waveguides is limited by bending losses in waveguides, which constrains the minimum practical radius of resonant ring modulators to bending radii below which the losses of conventional rib and strip waveguides are unacceptably high, as well as by the extended lengths of Mach-Zehnder modulators. Photonic crystal architectures, by contrast, promise smaller device sizes but suffer strict layout constraints imposed by the requirement that all waveguides must be oriented along the photonic crystal’s axes.

Silicon waveguide technology has encompassed several waveguide architectures such as rib and strip waveguides^12–14^, corrugated and slot waveguides^15–18^, and photonic band gap (PBG) structures^19–22^. Propagation losses as low as 0.7 dB/cm and 0.1 dB/cm at the wavelength of 1.55 µm set the standard for submicron strip and rib waveguides^14^. Until recently, PBG structures that can efficiently guide light and potentially serve as a platform for photonic integrated circuits were limited to photonic crystals (PhCs) platforms^21,22^. Newer classes of PBG structures include photonic quasicrystals (PhQCs)^23,24^, hyperuniform disordered solids (HUDS)^25–28^ and local self-uniform (LSU) structures^29^. In particular, stealthy hyperuniform disordered materials (see Methods) exhibit large PBGs which are both complete and isotropic. This allows light to propagate through the structure in the same fashion independent of direction - a feature impossible to achieve with PhCs and other waveguide architectures^5–8,30–33^. Additionally, the HUD platforms promise to address two key challenges associated with the cost-effective application of CMOS-compatible optical filters to optical interconnects: device density per unit chip area (as compared to rib and strip waveguide platforms) and improved layout flexibility^31–36^ (as compared to PhC platforms). Another advantage of the disordered systems when compared to their periodic counterparts is increased flexibility to locally-engineer the structure to create high-quality factors resonant defects, narrow waveguides with arbitrary curvatures and arbitrarily high-order power splitters^32–35^. Furthermore, since the disorder is a design resource in these structures, prospects for leveraging the structures’ tolerance to disorder may suggest new approaches to improving manufacturing yields of optical systems by employing HUDS platforms.

In this paper, we introduce a HUD platform as a locally engineered photonic system and a generic architecture for photonic integrated circuits. Our major goal is to demonstrate the HUD platform’s ultimate design flexibility and the built-in ability for seamless integration of pre-designed optical cavities and waveguides. The results for straight waveguides and filters reveal a great potential of silicon-on-insulator (SOI) HUDS photonic integrated circuits to be used in a host of applications at optical communication wavelengths^32,35^. Several resonator types such as in-line cavity resonator with and without an air-slot, and resonant cavities adjacent to the waveguide were examined in terms of compactness, quality factor, and temperature stability. We show that HUDS resonators as compared to standard micro-ring resonators (MRRs) or Mach-Zehnder interferometers (MZIs) exhibit less temperature-dependent resonant wavelength shift (TDRWS) and increased compactness. We also analyze the simulated performance of sub-volt and sub fJ/bit electrical modulation of a compact, yet high quality-factor PBG resonator when actively driven with ohmic contacts in a p+pinn+ configuration. The results reveal promising prospects for device density improvements of several times and a lower power consumption per bit compared to silicon optical modulators based on MRRs and MZIs.

## Methods

The structures analyzed in this paper are designed employing stealthy hyperuniform disordered network platforms. A point pattern is classed as hyperuniform, if for large *R*, the number variance σ(*R*)^2^ of the points contained within a spherical sampling window of radius *R* (in *d* dimensions) grows more slowly than the window volume, i.e., more slowly than *R*^*d*^ ^37^. As a consequence, in Fourier space, the structure factor associated with the hyperuniform pattern, *S*(***k***), approaches zero as |***k***| → 0 (omitting the forward scattering peak at zero wave number)^38,39^. Crystalline and quasicrystalline point patterns trivially satisfy this property. In the case of crystalline structures, the structure factor consists of a periodically ordered pattern of Bragg peaks, and its symmetry is inherited from the original point pattern (with an upper bound of six-fold symmetry in two dimensions). Quasicrystalline point patterns are aperiodic point patterns, whose Fourier spectrum consists of a dense set of k-space peaks (obeying the hyperuniformity constraint of *S*(***k***) → 0, when |***k***| → 0) without any upper limit on their rotational symmetry (*n*-fold symmetric quasicrystalline point patterns, with *n* - an arbitrarily large integer, are possible). For the point patterns considered in this work, we further constrain the disorder to stealthy patterns for which the structure factor *S*(***k***) is isotropic and precisely equal to zero for a finite range of wavenumbers 0 ≤ *k* ≤ *k*_C_, for some positive critical wavevector, *k*_C_^38^. Hyperuniform photonic materials are then constructed by decorating a hyperuniform stealthy point pattern with dielectric materials according to the protocol introduced in ref. ^25^.

**Figure 1:**
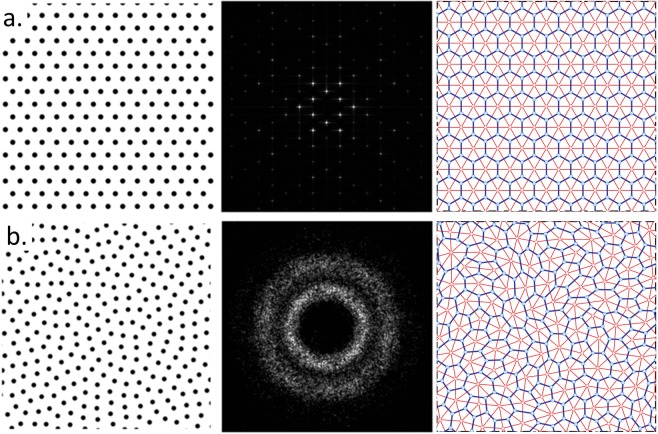
(**a**) Left to right: triangular lattice, its Fourier spectrum and the dual tessellation protocol which yields a honeycomb network. (**b**) Left to right: stealthy hyperuniform point pattern, its Fourier spectrum, and the dual tessellation protocol which transforms it in a trihedrally coordinated disordered network.

The hyperuniform disordered wall-network structures used in this study were designed by employing centroidal tessellations of hyperuniform point patterns to generate a “relaxed” dual lattice, a connected network structure whose vertices are trihedrally coordinated. As shown in Fig. 1, the protocol for generating these networks consists of Delaunay’s triangulating the hyperuniform point pattern and connecting the center of mass of Delaunay’s triangles to form polygonal cells with walls of finite width^24,25^. Optimally designed cavities and waveguides were then designed into this HUDS environment, using a 1/*r*^4^ potential to relax mismatched boundaries, where *r* is the average separation distance between scatterers. Design-optimization using full vectorial 3D FDTD Lumerical software (together with in-house developed simulation codes) was employed, leveraging a TE photonic band gap with zero density-of-states centered at around 1.55 μm wavelength.

HUDS waveguides and resonant defects were then fabricated using electron beam lithography and inductively-coupled plasma reactive ion etching at the University of Washington’s Nanofabrication Center^40^. Standard SOI wafers with 220-nm-thick crystalline silicon layer (on a 2 μm thick buried oxide layer) were used. HUDS devices with different average lattice spacings in the 473–500 nm interval, filling ratio covering from 37% to 43%, and wall widths of 120–200 nm were chosen aiming for high compactness, good temperature stability, and an isotropic photonic bandgap in the 1.5–1.6 µm wavelength range. The optimal PhC cavity, embedded in hyperuniform disordered environment, featured an average lattice spacing and filling ratio of 420 nm and 55%, respectively. Fully-etched focusing sub-wavelength grating couplers (lined up in an array with 127 μm spacing) were used at the input/output of the waveguide to provide efficient coupling of light from/to the single-mode optical fibers used for testing^35^. The measurements were performed in the wavelength range of 1.50–1.58 μm using an automated measurement setup as described in ref. ^41^. The continuous laser power was up to 15 dBm (32 mW) at 1550 nm, which was relatively low to observe any presence of optical nonlinearities in our measurements.

Next, a silicon photonic optical modulator employing HUD embedded cavities cavity was designed aiming for compactness and minimal energy per bit modulation. The modulator features both low- and high-dose doping regions (for ohmic contacts formation) targeting doping densities of phosphorus and boron ions of n = p = 5 × 10^18^ cm^−3^ and n+ = p+ = 1 × 10^19^ cm^−3^, respectively. Distances between the n and p regions, and between the aluminum electrodes were equal to 4.4 and 10 µm, respectively, while the widths of the n and p regions were 2.5 µm. The bias arrangement was to ground the p region while applying a negative voltage to the n region. The arrangement is known as a forward bias configuration, in which the pin diode acts as a variable resistor for voltages above a threshold voltage, because the resistance of the intrinsic region decreases with increasing current^42,43^. Full 3D simulations of both the optical performance and electron-hole dynamics were performed using a commercial-grade simulator of optical propagation based on the finite-difference time-domain method^44^ and a device simulator that self-consistently solves the Poisson and drift-diffusion equations in the active device^45^.

## Results

**Figure 2:**
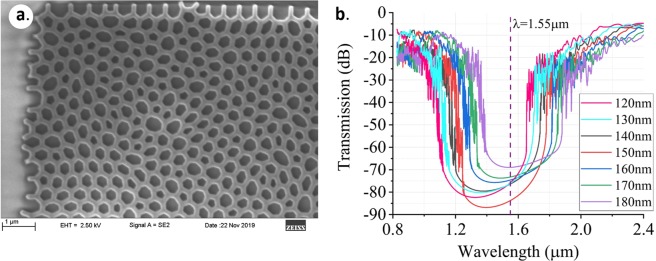
(**a**) Scanning electron micrograph image of a fabricated SOI HUD network structure with a wall width of 140 nm. (**b**) Simulated transmission results show that the position and width of the bandgap for the HUDS network with an average lattice spacing of 500 nm is tunable by varying the wall widths.

Figure 2(a) shows a scanning electron micrograph (SEM) image of a HUD network fabricated using electron-beam lithography on a 220-nm-high silicon-on-insulator wafer. The average spacing separating the centers of the network cells is 500 nm and the wall width is 140 nm. FDTD simulation results of the transmission spectrum for TE polarized light through hyperuniform networks with an average separation of 500 nm and various wall widths are shown in Fig. 2(b). As shown previously^25^, these networks possess wide TE polarization bandgaps, with a relative gap width of 25% of the central gap frequency. Also, the central wavelength of these bandgaps can be tuned by modifying the wall width of the HUDS. Since the bandgaps are wide, for wall widths ranging from 120 nm to 180 nm, the wavelength range of 1.50–1.58 µm can be easily covered. This property makes such networks a particularly well-suited platform for photonic circuit design, allowing straightforward integration of HUDsian devices with the full ecosystem of conventional rib and strip-waveguide components.

**Figure 3:**
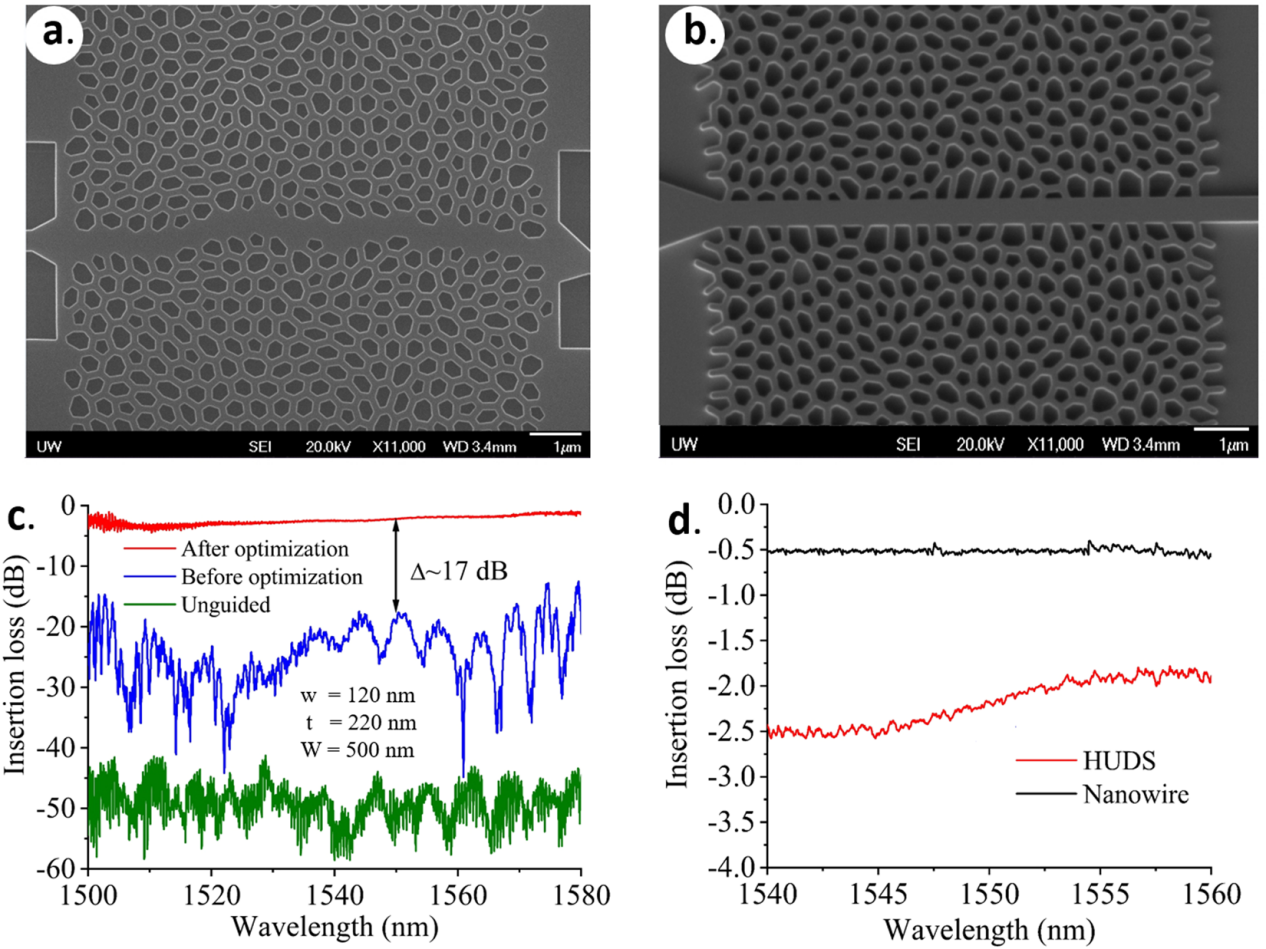
(**a**) SEM image of a fabricated SOI HUD waveguide by simply skipping a row of etched air holes. (**b**) SEM image of a fabricated SOI HUD waveguide (including design optimization). (**c**) Experimentally measured transmission spectrum comparing performance of HUD waveguide before (**a**) and after (**b**) design optimization shows a 17 dB improvement. A flat transmission spectrum across a large range was achieved after waveguide optimization. (**d**) Experimentally measured transmission spectrum comparing the coupling losses of the optimized HUD waveguide and a silicon strip waveguide. Insertion losses of ~2–3 dB were obtained due to the input/output coupling loss between HUD waveguide and the rest of the devices. Here, w represents the uniform width of the network walls, t is the waveguide thickness (height), and W is the average cell separation and also the fixed width of the waveguide channel in (**b**). The label ‘unguided’ in Fig. 3(c) refers to the HUDS structure without a waveguide channel embedded in it.

Next, waveguides were initially designed as a series of in-line defects by simply substituting one row of polygon-shaped air cells along desired paths with filled silicon. Figure 3(a,b) show SEM images of fabricated 220 nm thick SOI HUD waveguides. The intrinsic isotropy of HUDS allows for a variety of optimization approaches to be deployed in order to increase the transmission through the waveguide channel^32,33^. Here, to minimize backscattering losses, we performed a simple optimization of the waveguide structure in Fig. 3(a) by setting the waveguide width (500 nm) and wall width (120 nm) constant, and performing an automatic adjustment of the adjacent silicon walls to be nearly perpendicular to the waveguide channel as shown in Fig. 3(b). This one-step optimization substantially reduced the initially high backscattering loss of >3 dB/mm to 1.3 dB/mm at 1550 nm wavelength. Both simulations and experiments confirmed that the silicon HUDS network structures exhibit TE photonic band gaps, covering the 1.50 μm to 1.58 μm wavelength range for the chosen wall width and that the guiding mechanism is dominated by the presence of the photonic bandgap. Figure 3(c) shows the measured transmission spectrum through HUD waveguides before and after optimization, as well as the transmission in the absence of the waveguide channel. A 17-dB improvement at around 1550 nm associated with the above optimization was experimentally verified. A high transmission profile in the wavelength range of 1.54–1.58 μm was also observed in Fig. 3(d), where the HUD waveguide after optimization is compared with a Si strip waveguide (nanowire) to measure the coupling losses. In order to accurately measure the difference in coupling losses between the strip and the HUDS waveguide, we have designed, fabricated and measured 10 µm long devices so that the optical transmission through HUDS waveguide is similar to that through a 500 nm wide silicon strip waveguide. The results in Fig. 3(d), demonstrate that the total coupling losses between the HUD and nanowire waveguides are around 2 dB.

**Figure 4:**
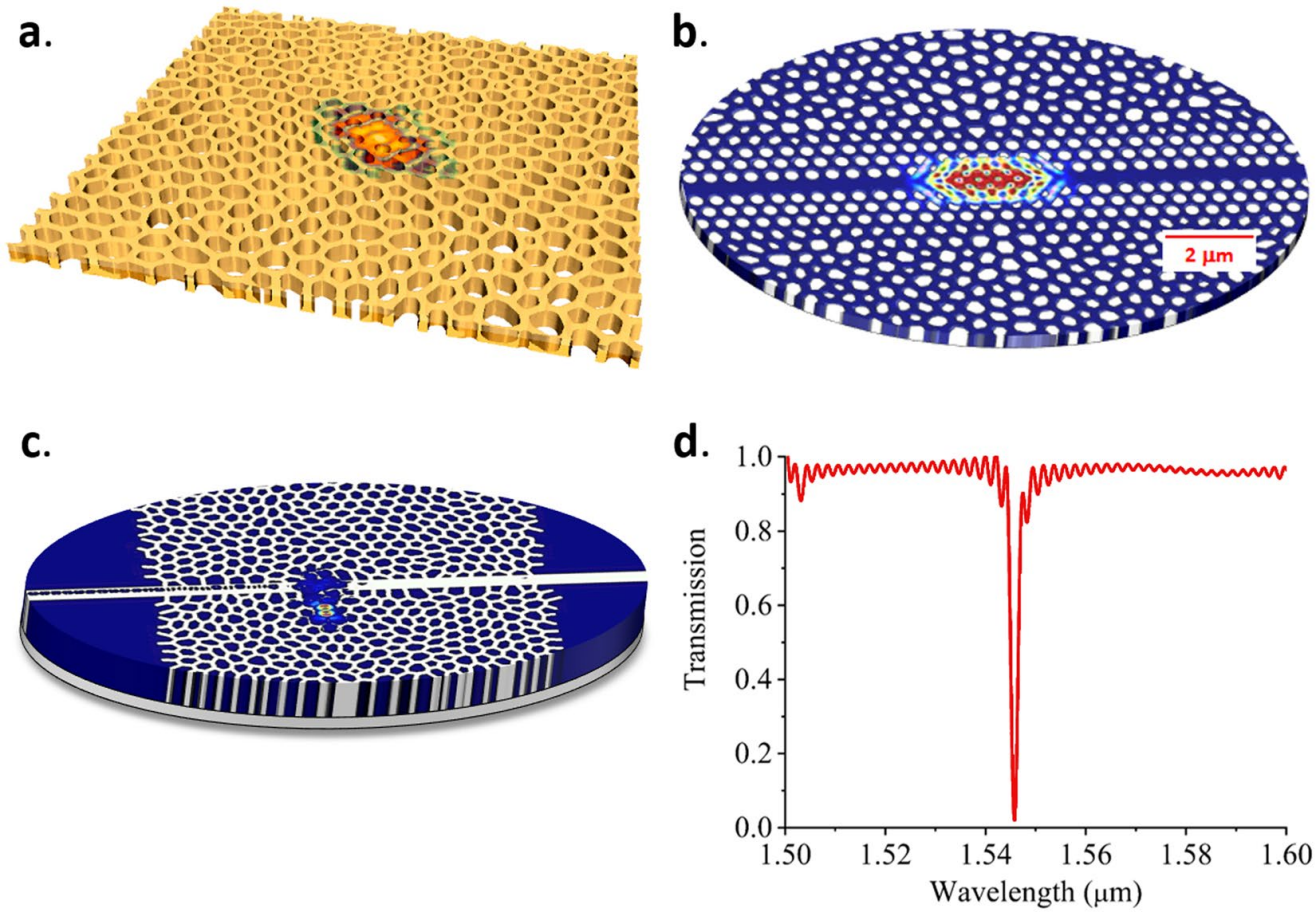
(**a**) Simulated mode field profile for a cavity with a Q-factor larger than 20,000 in a HUDS slab with TE polarization PBG. (**b**) Simulated mode field profile of the HUDS-cladded photonic crystal cavity with a Q factor of 1 million. (**c**) Simulated mode field profile of the resonant mode of a HUDS-based cavity-waveguide filter and (**d**) its simulated transmission spectrum.

**Figure 5:**
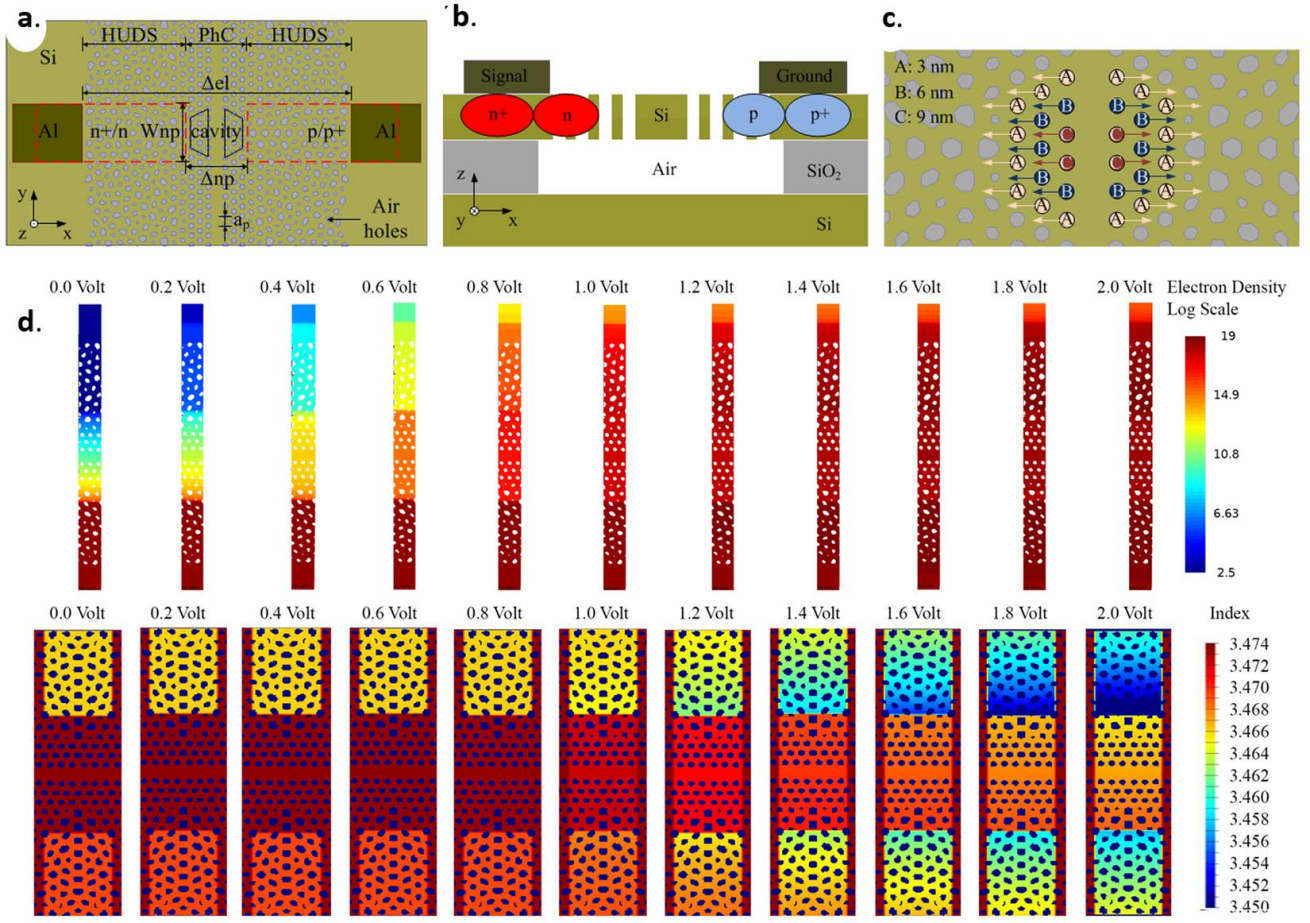
(**a**) A schematic of resonant modulators clad with HUDS in p+pinn+ configuration. The top view illustrates waveguide-coupled cavity clad with HUDS, and positions of doping regions. (**b**) Side view of the device illustrating approximate distributions of p (Boron) and n (Phosphorus) dopants. (**c**) HUDS resonant cavity design. (**d**) Top: Pseudo-color display of simulated electron density (log scale in C·cm^−3^) and bottom: the pseudo-color display of simulated index of refraction distribution (linear scale) for the p+pinn+ device as a function of the magnitude of the applied voltage.

In the following, we demonstrate that HUD platforms advantageously support a rich set of new resonator designs including resonant cavities with symmetries that are not available in photonic crystal structures^33,34^. The mode profile of such a cavity is shown in Fig. 4(a) and features Q-factor larger than 20,000. The versatility and flexibility of the HUD platform are evident as it allows not only for novel types of cavities and waveguide designs with no analog in conventional periodic architectures, but also for seamless integration of state-of-the-art designs developed for periodic structures. In the following, we focus on designs which integrate well-established high-Q PhC cavity designs^42^ in HUDsian claddings. Figure 4(b) shows the resonant mode field profile for a dispersion adapted PhC cavity embedded in a HUDs surround. The cavity is defined by shifting the holes around the center of the waveguide, following the pattern in Fig. 5(c), whereas the waveguide is defined by three rows of periodically arranged holes, which transition in a seamless manner into the HUDsian cladding. Despite the minimal number of periodic rows employed, the cavity maintains a very high-quality factor. The advantage of our approach is that any other types of devices can be integrated with minimal effort on the same platform with a freeform HUDsian cladding providing the needed insulation among the various components. As shown in ref. ^42^, resonators made of this type of PhC cavities, in general, have high Q factor and small footprints. Figure 4(b) shows that the optical mode in HUDS embedded PhC cavity is also tightly confined within 2–3 µm in the direction perpendicular to the waveguide while maintaining a quality factor of 1 million. Along the direction of the waveguide, the length of the barrier waveguides, which bridge the cavity and the input/output waveguides, determines the actual device footprint and packing density. The shorter the barrier waveguides are, the lower the Q factor is. Our simulations show that even when the HUD device footprint is about 12 μm^2^, the Q factor is still as high as 5 × 10^4^.

Due to a large thermo-optic coefficient of silicon material (1.86 × 10^–4^ K^−1^), environmental changes may cause significant device performance degradations in silicon photonic integrated circuits. MRRs are very sensitive to ambient temperature fluctuations. A TDRWS of about 0.1 nm per degree temperature is hard to be avoided^46^. Due to their bandgap, PBG cavities may provide a solution to this problem because the optical mode can be confined in air or silica which has an order of magnitude lower thermo-optic coefficient (10^–5^ K^−1^) than silicon. Our simulations show that PBG resonant cavities, both regular PhC cavities and the HUDS embedded cavities, featuring a 100 nm wide rectangular air slot along the waveguide can reduce the TDRWS to 0.04 nm per Kelvin. With an air slot, HUDS embedded PhC cavities can still have Q factors close to 10^4^. We have also investigated HUD resonant filters consisting of a point defect and a nearby, adjacently coupled waveguide as shown in Fig. 4(c). Simulation of a typical transmission spectrum shown in Fig. 4(d) exhibits a high extinction ratio of approximately 20 dB. Benefitting from the large PBG of the HUD network, all these HUDS-based cavity designs generally provide higher quality factors in a smaller footprint than micro-ring resonators^46–57^.

Next, we analyze an electrically-controlled optical modulator, featuring an air-bridged resonant cavity in a HUDS structure in p+pinn+ configuration. A schematic of the structure is shown in the Fig. 5(a–c). The high-Q (~3 × 10^6^) photonic crystal in-line cavity design chosen for this demonstration features a resonant cavity formed by very small translational shifts of holes by 3, 6, and 9 nm (Fig. 5(c)), respectively, as described in ref. ^42^. As emphasized before, our main goal here is to demonstrate the versatility of the HUD platform to seemingly integrate a variety of optical components while maintaining their state-of-the-art performance. Figure 5(d) top shows the electron distribution density as a function of the bias voltages for a stripe running between the two Al-electrodes and going through the center of the cavity. Correspondingly, Fig. 5(d) bottom shows the local refractive index distribution as a function of the biased voltages. It is clear that both the electron distribution density and the local refractive index can be easily tuned by small applied voltages.

**Figure 6:**
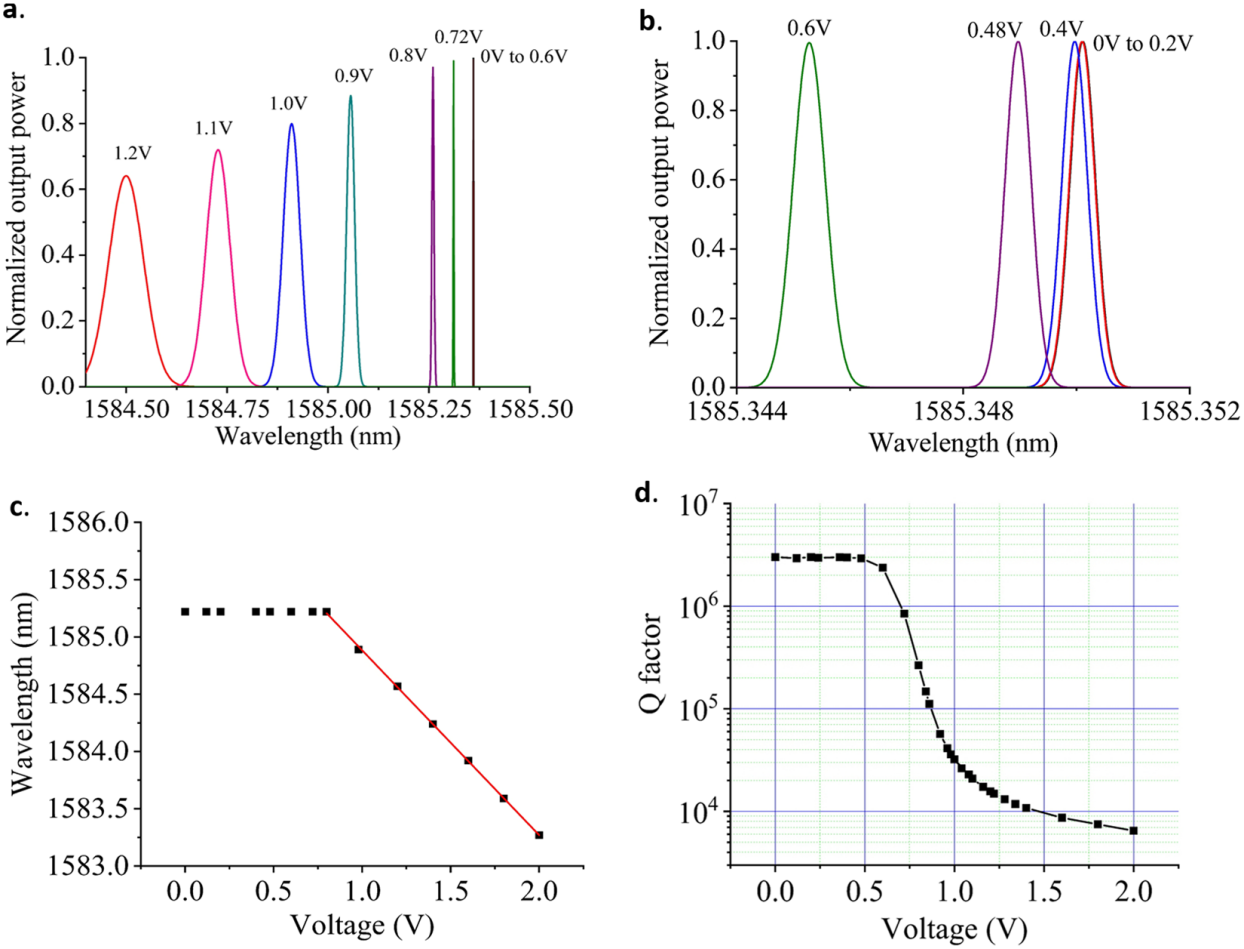
(**a**) Resonant wavelength shift as a function of the applied voltage for a voltage range from 0 to 1.2 V. (**b**) Resonant wavelength shift as a function of the applied voltage for a voltage range from 0 to 0.6 V. (**c**) Resonant wavelength peak position as a function of applied voltage illustrating linear and steep resonant peak shifts for voltages higher than 0.8 V. (**d**) The corresponding quality factor of the resonant wavelength peaks as a function of the applied voltage.

Figure 6(a) demonstrates that the transmittance spectrum shifts towards a shorter wavelength when a forward bias is applied. This implies that the refractive index of silicon has been reduced, as expected for the plasma dispersion effect^58^. As shown in Fig. 6(b), an applied voltage as small as 0.48 V is sufficient to shift the resonance peak away from the 0 V peak more than their widths, separating them sufficiently to assure a 10 dB on/off ratio. Furthermore, for voltages up to 0.8 V, the optical Q factor of the resonant wavelength peak can remain larger than 10^5^, indicating little change in the corresponding full width half maximum (FWHM) line width <0.016 nm (see Fig. 6(b)). We thus predict 0.48 V to be the threshold voltage to operate this modulator at a 10 dB on/off ratio. For voltages above 0.7 V, we found the voltage dependence of wavelength to be 1.6 nm/V, as shown in Fig. 6(c), which is comparable to modulators based on MRRs^49,51^. Therefore, this modulator can work at 0.48 V and an AC drive voltage about 70 mV (peak to peak) to turn the signal on and off. Both the threshold voltage and anticipated peak-to-peak drive voltage is 2–3 times smaller than what has been reported in “ultra-low voltage” MRRs^51^. Correspondingly, anticipated power consumption of 0.4 fJ/bit for a 6 dB on/off ratio was calculated, following the methodology described in^42^. This low power operation is due to the small size and high-Q of the resonant cavity. Figure 6(d) shows the quality factor of the resonant peaks as a function of applied voltages. The quality factor remains around 1 M at the applied voltages discussed above.

**Figure 7:**
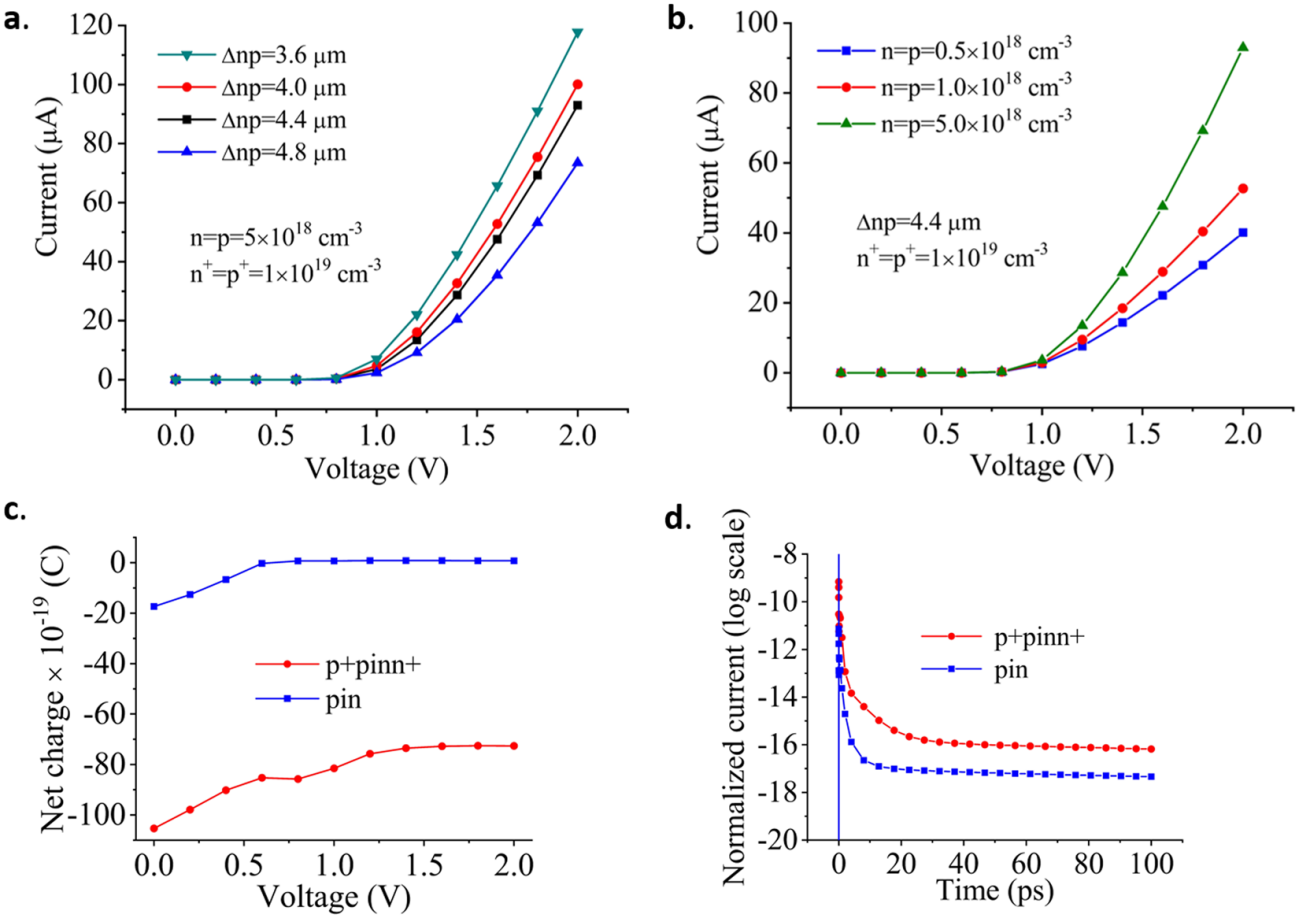
(**a**) I-V characteristic as a function of the distance between doping regions for constant doping densities. (**b**) I-V characteristic as a function of doping densities at a constant distance between doping regions. (**c**) Net charge as a function of the applied voltage. (**d**) The transient analysis provides time constants of 0.313 ns and 0.148 ns, for pin and p+pinn+ configuration, respectively, corresponding to a bandwidth of 0.508 GHz and 1.07 GHz.

In this analysis, we have used a low dose doping of phosphorus and boron ions with densities of n = p = 5 × 10^18^ cm^−3^ together with the high-dose doping regions (n+ = p + = 1 × 10^19^ cm^−3^) for ohmic contacts formation. A 4.4 µm distance between the doping regions was chosen to provide relatively high resistance (Fig. 7(a)) and to reduce the optical losses caused by doping. Figure 7(b) shows the current/voltage characteristic as a function of doping densities at a constant distance between doping regions. A doping density of n = p = 5 × 10^18^ cm^−3^ featured relatively high resistance which was enough to provide efficient optical modulation. We also analyzed a “pin” device configuration (without high doping densities for ohmic contact formation). Figure 7(c) shows a few times lower absolute net charge of the same device in p + pinn + configuration which corresponds to a lower capacitance of this device. We performed transient analysis (Fig. 7(d)) by applying a square voltage pulse of 70 mV, which revealed time constants of 0.313 ns and 0.148 ns, for the pin and p+pinn+ configuration, respectively, corresponding to bandwidths of 0.508 GHz and 1.07 GHz. Although much slower than MRR and MZI based modulators, the modulation speed of about 1 GHz could be improved to significantly higher values by exploiting a similar approach of embedding MZI configurations into HUD surround and operating in the reverse bias regime.

## Discussion

Our simulation and experimental results of HUD-integrated devices demonstrate the functionality of HUDS as a flexible and compact platform for silicon photonic integrated circuits. We demonstrate propagation losses of 1.3 dB/mm for partially optimized HUD waveguides, comparable to propagation losses of PhC waveguides. Additional reduction of the propagation losses can be achieved by improvements in the fabrication process, further optimization of the transition between the HUDS and the strip waveguides, use of wider waveguides and post-fabrication treatments (thermal oxidation and removal of the SiO_2_ layer underneath)^55,59–61^. It is worth noting that, even if the HUDS holes are filled with SiO_2_, the silicon HUDS platforms can still have a sizeable TE polarization bandgap with a relative width of about 15%. The position of this bandgap can be tuned by varying the HUDS wall widths and/or the average HUDS lattice spacing. Therefore, HUDS waveguides and devices can work well when SiO_2_ cladding is used. Although electron beam lithography was used to fabricate devices reported in this paper, optical lithography can also be used to fabricate HUDS network structures with wall widths of 120–180 nm^62^.

Our results demonstrate the potential of integrating various resonator types such as in-line HUDS resonant cavities or adjacently-coupled resonator/waveguide structures in HUDS surrounds. We have also showed that HUDS can be employed to facilitate light confinement in predefined PhC resonant cavities and enhance their temperature stability. Since a temperature-dependent resonant wavelength shift depends on the resonator size and coupling strength to the nearby waveguide^46,55^, we suggest that the TDRWS reduction of a HUDS resonator is related to the relatively small defect size and weaker light interaction with silicon than in the case of MRRs. Furthermore, a HUDsian cladding allows for a much richer variety of cavity designs (see Fig. 4 and examples in refs. ^26,32–34^). which offer increased control over the engineering of cavity field spatial distributions and hence further opportunities to decrease the TDRWS. In all cases, the size of the cavity was much lower than the typical area of a standard MRR implying higher compactness of the HUDS platform for PICs applications.

**Figure 8:**
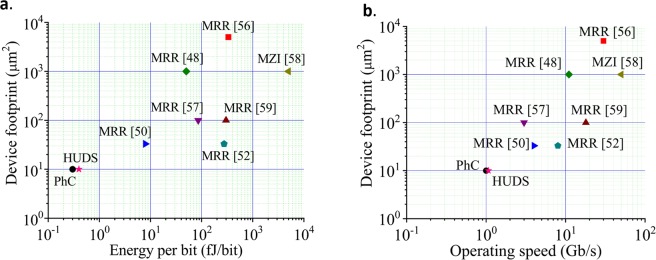
Device footprint as a function of (**a**) energy per bit and (**b**) operating speed for state-of-the-art silicon optical modulators. HUDS-based modulator (red star) and photonic crystal-based modulator (black circle) designs share with photonic crystal modulators the same orders of magnitude improvement in compactness and energy efficiency as compared to modulators based on MRRs and MZIs.

As shown in Fig. 8(a), optical modulation using the HUDS platform provides device density improvements of several times and lower energy per bit compared to silicon optical modulators based on MRRs and MZIs. For comparison, we have also analyzed a “pin” structure. Simulations of the voltage dependence of wavelength shifting, Q-quenching, and corresponding optical cavity lifetimes show similar behavior for the pin and the p+pinn+ device designs. However, we find that the operating speed of a pin modulator is up to two times lower compared to a configuration with ohmic contacts. In the current configuration, operating speed slightly exceeds 1 Gb/s (Fig. 8b), which is still an improvement to similar designs described in ref. ^42^. In order to achieve higher operating speed, it is necessary to use MZI configurations in the reverse bias regime, already proven to enable up to 32 Gb/s operation for PhC based modulators^63–66^. As indicated in Fig. 6(a,b), threshold voltage of 0.48 V operates the current modulator structure at a 10 dB on/off ratio. However, the worst-case estimation of signal broadening due to factors such as sidewall roughness (Q factor may be reduced to 10^4^), a DC voltage of 0.8 V and with 70 mV (peak to peak) AC voltage can be used to operate the device at a 6 dB on-off ratio using a square wave. Both voltages are still lower than what has been reported for “ultra-low voltage” MRRs. Our HUDS modulators could provide the following advantages over MRR and MZI-based modulators: a smaller footprint, increased device density, lower energy per bit (as shown in Fig. 8), and better temperature stability. These electrically controlled optical modulators are designed to be fabricated in the same SOI material system using fully-CMOS-compatible fabrication processes as are being used to fabricate MRR devices currently undergoing very rapid commercialization efforts, and their layouts are not constrained to follow the axes of photonic crystals.

## Conclusion

In conclusion, we introduced a HUDS silicon photonics platform as both local and global engineered environment for PIC applications by experimentally demonstrating the lithographic fabrication of HUDS waveguides in silicon-on-insulator for operation over the range of ~1.5 to 1.6 microns. The intrinsic isotropy of these novel disordered PBG materials demonstrates the potential for photonic device design by offering compactness, low-power consumption, and improved temperature stability combined with unprecedented design freedom not limited by crystalline structures and periodicity. The disordered character of hyperuniform materials makes them less sensitive to fabrication errors that create randomly distributed disorder, as compared to their periodic counterparts. The resonant devices based on HUD demonstrate a clear ability to guide light and localize light in the infrared regime with low loss and lower temperature-dependent resonant wavelength shift than that of the standard silicon micro-ring resonators. Design optimization of an optical modulator based on an inline-coupled resonant PBG cavity predict sub-volt and sub-fJ/bit electrical modulation when driven with ohmic contacts in a p+pinn+ configuration. Temperature-stabilized, low-loss, compact and energy-efficient HUD devices, therefore provide new building blocks for the design of more complex systems featuring both passive and active devices, enabling new opportunities for cost-effectively increasing the data rates supportably in SOI, as well as in other semiconductor material platforms, and for other applications.

## Data Availability

The data underlying the findings of this study are available without restriction^67^.
